# Kras mutation is a marker of worse oncologic outcomes after percutaneous radiofrequency ablation of colorectal liver metastases

**DOI:** 10.18632/oncotarget.19806

**Published:** 2017-08-02

**Authors:** Waleed Shady, Elena N. Petre, Efsevia Vakiani, Etay Ziv, Mithat Gonen, Karen T. Brown, Nancy E. Kemeny, Stephen B. Solomon, David B. Solit, Constantinos T. Sofocleous

**Affiliations:** ^1^ Section of Interventional Radiology, Department of Radiology, Memorial Sloan Kettering Cancer Center, New York, NY 10065, USA; ^2^ Department of Pathology, Memorial Sloan Kettering Cancer Center, New York, NY 10065, USA; ^3^ Department of Epidemiology and Biostatistics, Memorial Sloan Kettering Cancer Center, New York, NY 10065, USA; ^4^ Department of Medicine, Memorial Sloan Kettering Cancer Center, New York, NY 10065, USA

**Keywords:** percutaneous radiofrequency ablation, colorectal liver metastases, Kras mutation, oncologic outcomes

## Abstract

**Background:**

Kras mutation has been associated with shorter overall survival and time to disease recurrence after resection of colorectal liver metastases (CLM). This study evaluated the prognostic value of Kras mutation in patients with CLM treated by percutaneous radiofrequency ablation (RFA).

**Methods:**

This is an IRB waived retrospective analysis of the impact of KRAS mutation status on oncologic outcomes after CLM RFA. The endpoints were overall survival (OS), local tumor progression (LTP) rates, and incidence of new liver, lung, and peritoneal metastases. Survival times were calculated using Kaplan-Meier methodology from the time of RFA.

**Results:**

The study enrolled 97 patients. Kras exon 2 mutation was detected in 39% (38/97) of patients. On univariate analysis, Kras mutation (P=0.016) (HR: 1.8; 95% CI: 1.1 – 2.9) was a significant predictor of OS and retained significance on multivariate analysis. Kras mutation was a significant predictor of new liver metastases (P=0.037) (SHR: 2.0; CI: 1.0-3.7) and peritoneal metastases (P=0.015) (sHR: 3.0; 95% CI: 1.2-7.2) on multivariate analysis. Kras mutation was a significant predictor of LTP after RFA of CLM ablated with margins of 1-5 mm (P=0.018) (SHR: 3.0; 95% CI: 1.2-7.7) with an LTP rate of 80% (12/15) versus 41% (11/27) for wild type.

**Conclusion:**

Kras mutation is a significant predictor of overall survival, new liver, and peritoneal metastases after RFA of CLM. A minimal radiographic ablation margin ≥ 6 mm is essential for local tumor control especially for mutant CLM.

## INTRODUCTION

Radiofrequency ablation (RFA) of unresectable colorectal liver metastases (CLM) has been associated with a 5-year overall survival (OS) rates of 31-48% [1–4], with a single cohort reporting a 10 year OS of 18% [3]. Studies have shown a relatively short recurrence-free survival and high overall recurrence rates for ablation when compared to historic hepatectomy outcomes [1, 2, 5–7]. Important predictors of oncologic outcomes include extrahepatic disease, high CEA level, >1 liver tumor, tumor size >3 cm, minimal ablation margin size under 5 mm around the tumor [1, 2, 5, 8], as well as markers of tumor viability and prolific activity from the ablated tumor [9–11].

Surgical series have reported the prognostic value of genetic markers [12]. Kras as well as BRAF mutation has been associated with higher incidence of disease recurrence and shorter OS after resection for colon cancer liver metastases (CLM) [13–15].

The aim of this study is to evaluate the prognostic value of Kras mutation as a predictor of overall survival, local tumor progression (LTP) and incidence of location specific metastases in patients undergoing percutaneous RFA for CLM.

## RESULTS

### Mutational status

Kras exon 2 mutations were detected in 39 % (38/97) of patients and included: G12D (32%), G12V (21%), G13D (21%), G12C (10%), G12A (7%), G12R (3%), G12S (3%), and G13C (3%) mutations. Braf mutation status was analyzed in 65% (63/97) of patients with only 8% (5/63) of patients harboring a mutation. The panel of 8 genes was analyzed in 33% (32/97) of patients. In these patients, extra mutations were seen in Nras 9% (n=3/33), PIK3CA in 13% (n=4/33), Akt1 (n=1/33), and ERBB2 (n=1/33).

### Overall survival

**Figure 1:**
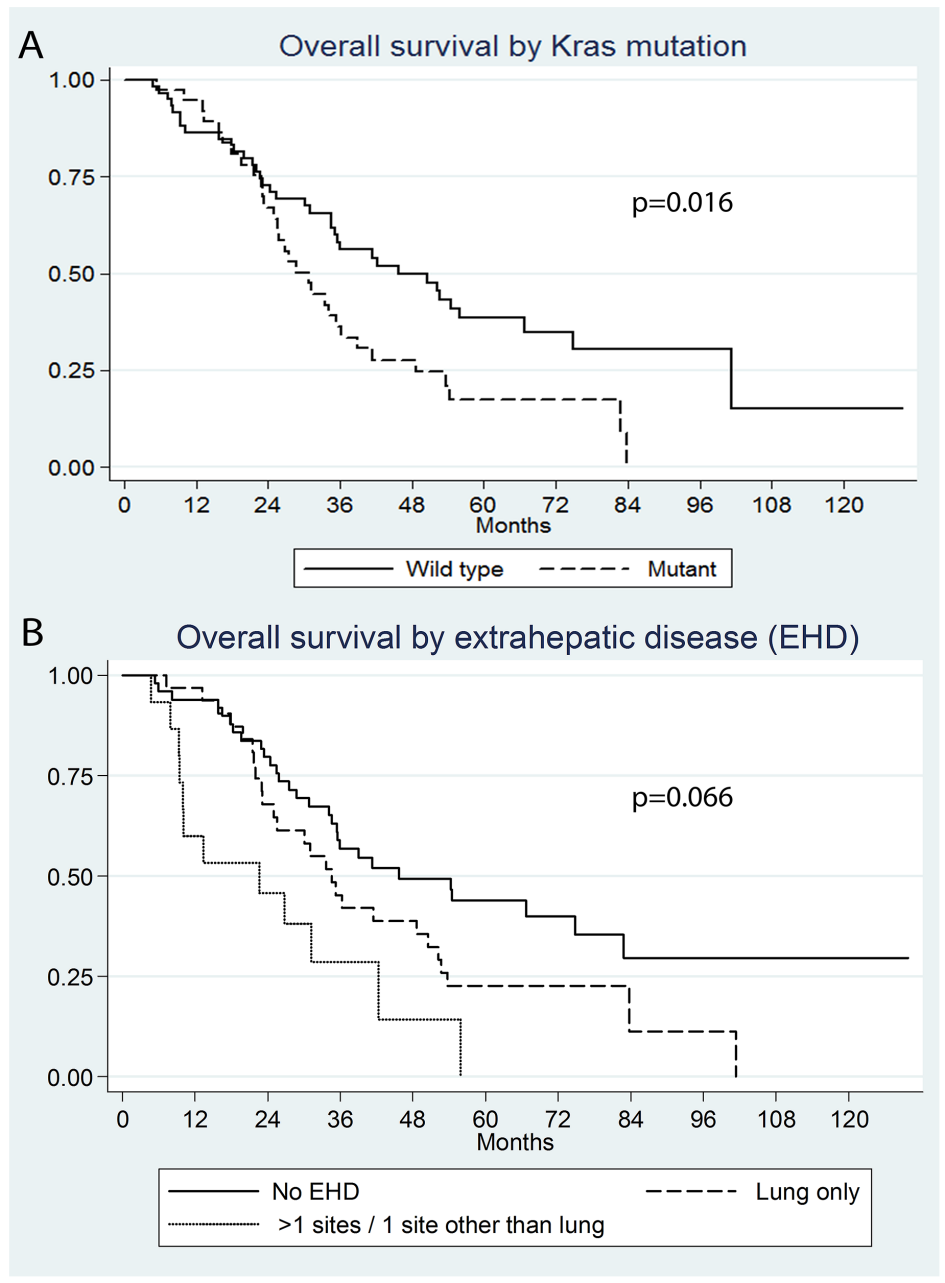
Kaplan Meier survival curves by **(A)** Kras mutation (P=0.016), **(B)** extrahepatic disease (>1 site/other site of EHD versus no EHD P<0.001, lung only EHD versus no EHD P=0.066)

The median OS was 35.5 months (95% CI: 30.8 – 48.6). The 1, 3, and 5 year OS rates were 89.6%, 48.5%, and 30.3% respectively. On univariate analysis (UV) significant predictors of shorter OS included: CEA level >30 ng/ml (P=0.038) (HR: 2.0; 95% CI: 1.0 – 3.8) (median OS of 41 versus 23 months), Kras mutation (P=0.016) (HR: 1.8; 95% CI: 1.1 – 2.9) (median OS of 46 versus 31 months) (Figure 1A), and >1 site/other site of EHD (P<0.001; HR: 3.8; 95% CI: 1.8 – 8.0) (median OS of 13.4 months versus 46 months for no EHD) (Figure 1B). Tumor size >3cm was only marginally significant (P=0.09) (HR: 1.7; 95% CI: 0.91-3.4). On MV, >1/other site of EHD and Kras mutation retained significance, whereas CEA >30ng/ml approached significance (Table 1).

**Table 1:** Multivariate analysis of predictors of overall survival

Predictor	P-value	Sub-hazard Ratio	95% CI
EHD >1 site / 1 site outside the lung	0.001	3.0	1.6 - 5.9
Kras mutant	0.009	2.0	1.2 - 3.3
CEA >30 ng/ml	0.056	1.9	0.98 - 3.7

## LTPFS

**Figure 2:**
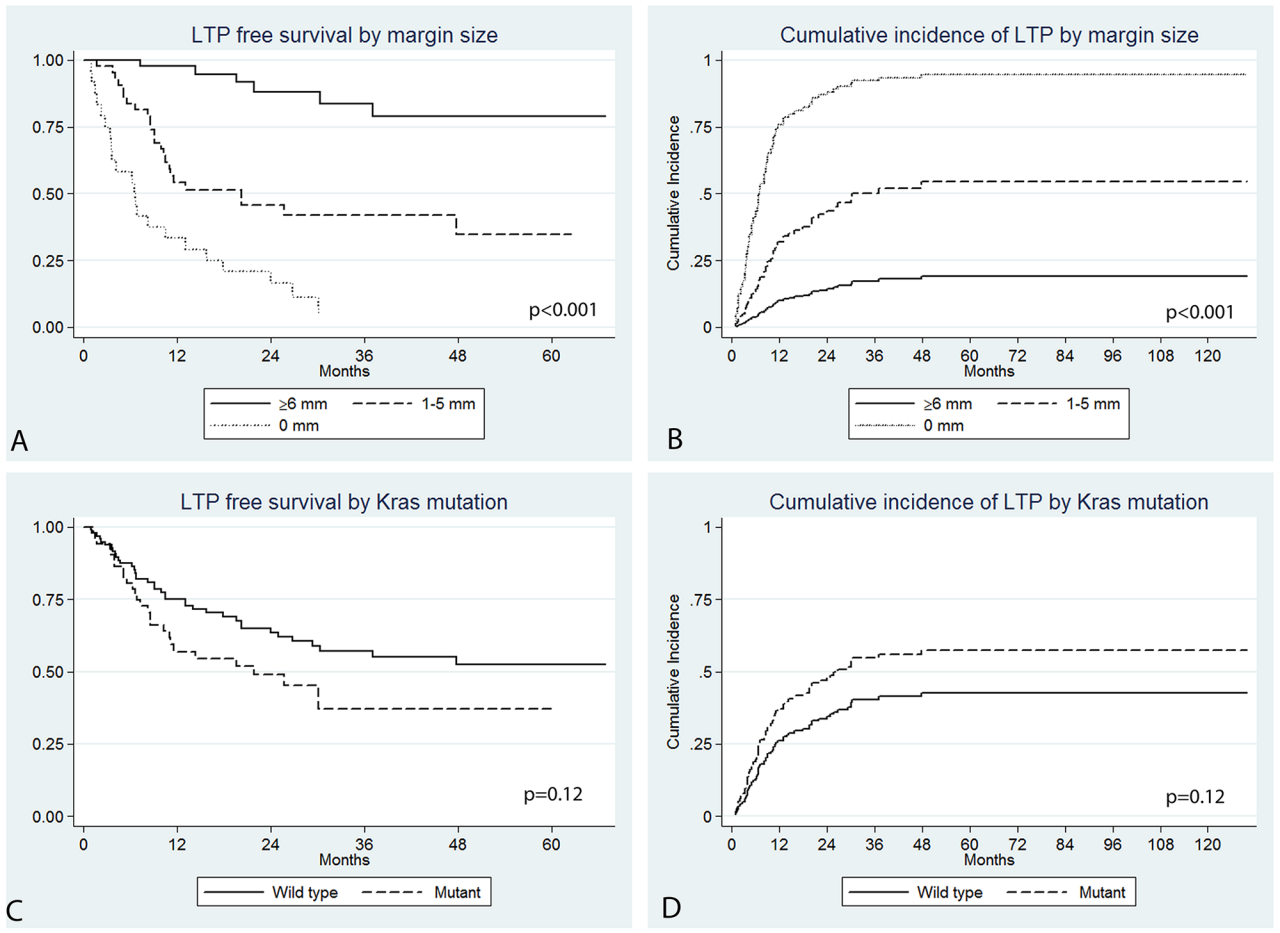
Kaplan Meier LTPFS **(A)** and cumulative incidence **(B)** curves by ablation margin size (P<0.001) and Kras mutation (P=0.12).

Technique effectiveness for the ablated target tumors was 95% (141/148). One of the failures was reablated, one was lost to follow up, while in the other five cases systemic chemotherapy or selective internal radiation therapy were the only available options due to the rapid development of new intra- and/or extrahepatic metastases (n=4) or new lesion near IVC not amenable to ablation (n=1). Median LTPFS was 34 (range: 4.7 – 129.8) months. The 1, 3, and 5 year LTPFS were 68.8%, 50.1%, and 47.3%. During the median follow-up period of 60.1 months, 43.9% of tumors (65/148) developed LTP. Almost all LTPs (96.9%; 63/65) occurred within 3 years post-RFA; 67.8% (44/65) during the 1^st^ year, 15.4% (12/65) during the 2^nd^ year, and 13.8% (7/65) during the 3^rd^ year. On univariate analysis predictors of shorter LTPFS were: tumor size > 3cm (P=0.001) (subhazard ratio, sHR: 2.9; 95% CI: 1.5 – 5.5) (LTP rate of 40% (52/130) versus 72% (13/18)), no history of prior liver resection (P=0.01) (sHR:2.1; 95% CI: 1.2 – 3.7) (LTP rate of 40% (49/123) versus 64% (16/25)), no history of prior HAIC (P=0.007) SsHR:2.0, 95% CI: 1.2 – 3.3) (LTP rate of 36% (33/92) versus 57% (32/56)), and ablation margin size (P<0.001) (Figure 2A-2B) (LTP rates of 92% (22/24) for 0 mm margin, 56% (24/43) for 1-5 mm margin, and 13% (6/46) for ≥6 mm margin).

There was a trend for Kras mutation to be associated with shorter LTPFS on univariate analysis (P=0.12) (sHR: 1.5; 95% CI: 0.89-2.7). The LTPFS rate for the Kras mutant versus wild group at 1, 2, and 3 years were 56.9% versus 75.1%, 49.0% versus 63.5%, and 37.0% versus 57.1% respectively (Figure 2C). The cumulative incidence of LTP for the Kras mutant and wild type tumors at 1, 2, and 3 years were 41.6% versus 24.1%, 48.3% versus 34.4%, and 56.8% versus 39.7% respectively (Figure 2D).

**Figure 3:**
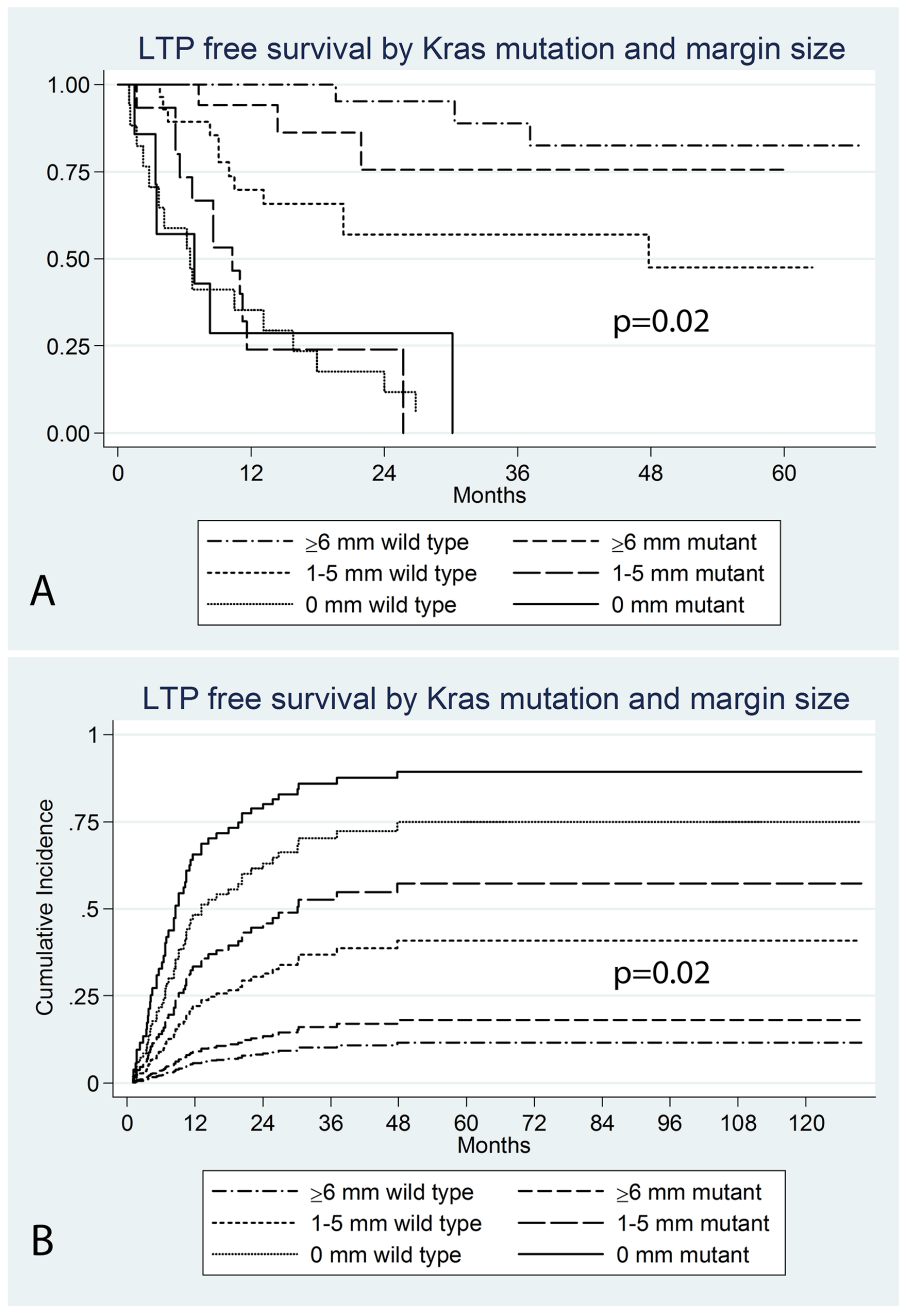
Kaplan Meier LTPFS **(A)** and cumulative incidence **(B)** curves by Kras mutation within ablation margin categories (p=0.02).

Analyzing the prognostic value of Kras mutation within each margin category revealed that the mutation was a significant independent predictor of LTPFS in tumors with a margin size of 1-5 mm (P=0.02) (sHR: 2.9; 95% CI: 1.2-7.3) (Figure 3); LTP rate for mutant CLM was double (80%: n=12/15) that of wild type CLM (43%: n=12/28). On multivariate analysis, only ablation margin size retained statistical significance (Table 2). LTP rates for each margin category according to mutation status are displayed in Table 3.

**Table 2:** Multivariate analysis of predictors of local tumor progression free survival

Predictor	P-value	Sub-hazard Ratio	95% CI
Ablation margin 0 mm (vs ≥6 mm)	<0.001	16.6	6.4 – 43
Ablation margin 1-5 mm (vs ≥6 mm)	<0.001	5.9	2.5 – 14
Size >3 cm	0.068	1.8	0.96 - 3.3
Kras mutant	0.11	1.7	0.89 - 3.2

**Table 3:** LTP rates according to margin size and Kras mutation

Margin + Kras status	P-value	Sub-hazard ration	95% CI	LTP rate
≥6 mm + wild type	Ref	Ref	Ref	3/28 (11%)
≥6 mm + mutant	0.5	1.7	0.3-8.4	3/18 (17%)
1-5 mm + wild type	0.01	5.1	1.4-17.7	12/28 (43%)
1-5 mm + mutant	<0.001	15.6	4.4-55.0	12/15 (80%)
0 mm + wild type	<0.001	22.9	6.5-81.4	16/17 (94%)
0 mm + mutant	<0.001	19.3	4.9-75.5	6/7 (86%)

### Site specific recurrence

#### New liver metastases

**Figure 4:**
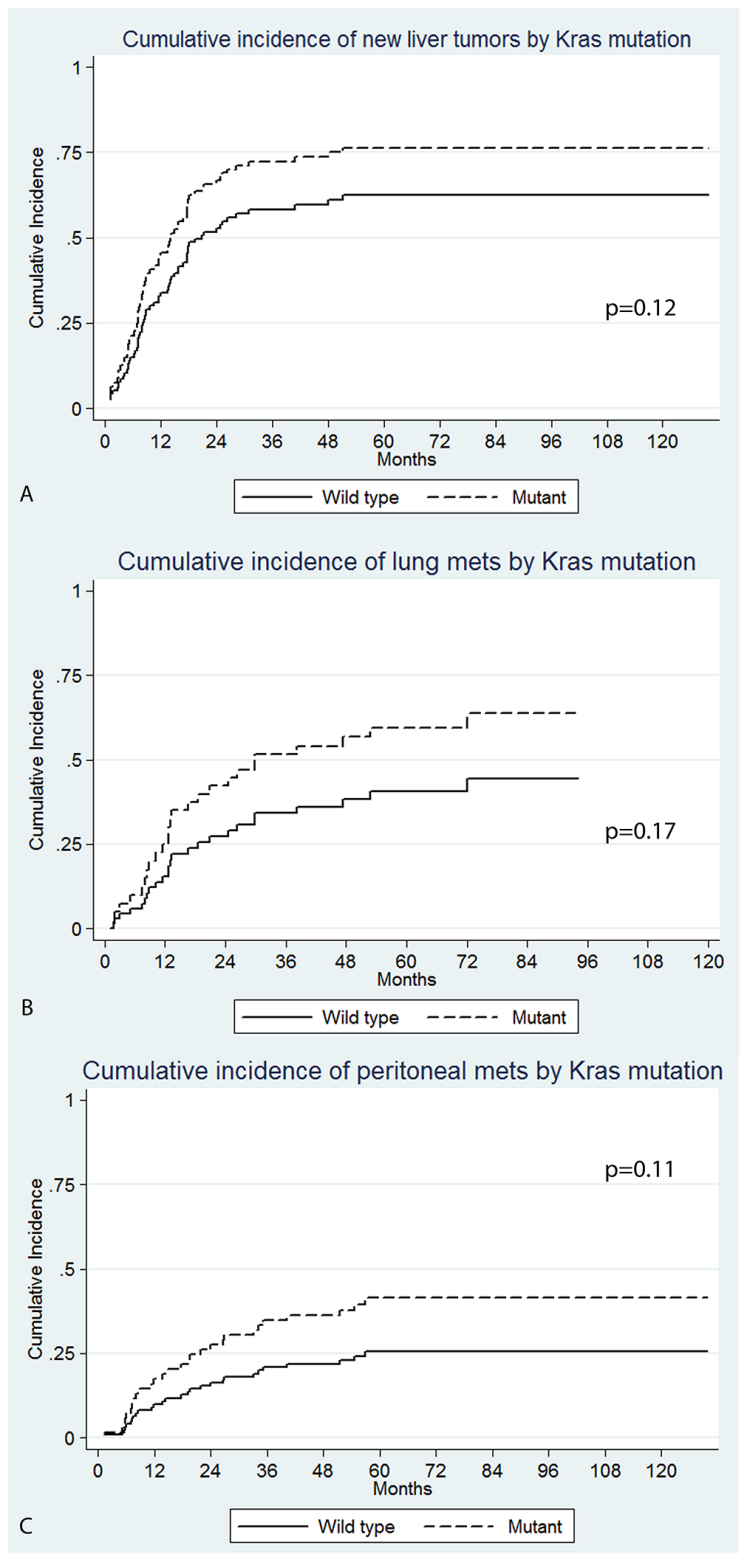
Cumulative incidence of site specific recurrence by Kras mutation; **(A)** new liver tumors (P=0.12 on univariate and P=0.037 on multivariate analysis), **(B)** lung (P=0.17), **(C)** peritoneal (P=0.11 on univariate and P=0.015 on multivariate analysis).

New liver metastases developed in 66% (64/97) of patients. A higher percentage of patients in the Kras mutant group developed new liver metastases (73.7%; 28/38) versus (61%; 36/59) in the wild type (P=0.2). There was a trend of shorter new liver metastases free-survival rate in the Kras mutant group at 1, 2, and 3 years; 55.9% versus 63%, 26.6% versus 43.9%, and 19.0% versus 36.1% respectively (P=0.12). The cumulative incidence of new liver metastases for the Kras mutant and wild type tumors at 1, 2, and 3 years were 43.0% versus 35.7%, 67.4% versus 53.1%, and 72.8% versus 58.5% respectively (Figure 4A); on univariate analysis (P=0.12) (sHR: 1.5; 95% CI: 0.90-2.4). On multivariate analysis, Kras mutation was an independent predictor of new liver metastases (P=0.037) (sHR: 2.0; CI: 1.0-3.7) (Table 4)

**Table 4:** Multivariate analysis of predictors of new liver tumors free survival

Predictor	P-value	Sub-hazard Ratio	95% CI
History of prior liver resection	0.020	2.5	1.1-4.9
EHD >1 site / 1 site other than lung	0.027	3.0	1.1-5.3
Ablation margin 0 mm (vs ≥6 mm)	0.017	2.5	1.2-5.1
Ablation margin 1-5 mm (vs ≥6 mm)	0.010	2.6	1.3-5.7
Kras mutation	0.037	2.0	1.0-3.7

#### New lung metastases

Fifty-three patients did not have detectable lung metastases prior to RFA. A higher percentage of Kras mutant patients developed new lung metastases (55%; n=12/22) than the wild type group (42%; 13/31) (P=0.37), however, this did not reach statistical significance. There was a trend for shorter lung metastases free-survival rate in the Kras mutant group at 1, 2, and 3 years: 63.4% versus 89.3%, 44.7% versus 73.7%, and 37.3% versus 61.1% respectively (P=0.17). The cumulative incidence of lung metastases in the Kras mutant group was higher than in the wild type patients at 1, 2, and 3 years: 32.8% versus 9.7%, 47.2% versus 23.1%, and 52.0% versus 33.4% respectively (Figure 4B). However, these differences did not reach statistical significance on univariate analysis (P=0.17) (SHR: 1.7; 95% CI: 0.79-3.8) (Figure 4). Patients who did not undergo a prior liver resection had a significantly shorter time to new lung metastases on univariate analysis (P=0.007) (sHR: 3.1; 95% CI: 1.4-7.1).

#### New peritoneal metastases

At the time of RFA 95/97 patients did not have any peritoneal disease. Peritoneal metastases developed in 29.5 % of patients (n=28/95). A higher percentage of patients in the Kras mutant group developed peritoneal metastases (n=14/37; 38%) versus (n=14/58; 24%) in the wild type (P=0.15). There was a trend of a lower peritoneal metastases free-survival rate in the Kras mutant versus the Wild type group at 1, 2, and 3 years; 79.6% versus 90.5%, 68.5% versus 82.3%, and 53.9% versus 76.1% respectively (P=0.11). The cumulative incidence of peritoneal metastases for the Kras mutant and wild type patients at 1, 2, and 3 years were 19.4% versus 8.8%, 28.1% versus 16.0%, and 36.8% versus 19.7%; (P=0.11) (sHR: 1.8; 95% CI: 0.87-3.8) (Figure 4C). On multivariate analysis, only Kras mutation maintained significance (P=0.015) (s HR: 3.0; 95% CI: 1.2-7.2). None of the other predictors reached statistical significance on univariate analysis.

#### New bone and brain metastases

Bone metastases developed in 14/97 (14.4%) of patients; 16% (6/38) in the Kras mutant and 14% (8/59) in the wild type. Brain metastases developed in 6/97 (6.2%) of patients; 5% (2/38) in the Kras mutant group and 7% (4/59) in the wild type. These numbers were too small for inferential statistical analysis.

## DISCUSSION

Disease control and OS for Kras mutant patients treated by RFA for CLM can be expected to be relatively lower than those with Kras wild type, similarly to what was shown after resection [14–19]. In this study, Kras mutation was an independent predictor of shorter OS, time to new liver and peritoneal metastases. Kras mutant tumors ablated with 1-5 mm margins had double the LTP rate of wild type. These findings suggest that the Kras status can be used as a prognostic biomarker of oncologic outcomes post-RFA for CLM.

Kras mutation is associated with shorter overall survival and higher incidence of metastatic spread. Updated results from the CRYSTAL and FOCUS studies reported shorter overall survival for patients with Kras mutation [20, 21]. A large retrospective study similarly reported Kras mutation as an independent predictor of shorter overall survival in patients with metastatic colorectal cancer [22]. The cumulative incidence of metastases at 2 years for sites that were not involved at the time of diagnosis was higher for Kras mutant patients [22]. This was significant for lung (P=0.001, 32.5% versus 19%), bone (P=0.024; 8.8% versus 4.4%), and brain metastases (1.4% versus 0.2% P<0.01), but not for liver metastases (P=0.78; 12% versus 14.3%). Another study for patients undergoing hepatic arterial infusion chemotherapy plus systemic chemotherapy after resection of liver metastases noted that Kras mutation was an independent predictor of shorter recurrence free survival at 3 years (30% vs 46%, P=0.005). Similarly, the 3 year cumulative incidence of lung (58% vs 33% P<0.01), bone (13.4% vs 2% P<0.01), brain (14.5% vs 2% P=0.05), and liver (47.3% vs 20% P=0.1) metastases was higher in the Kras mutant patients [15].

A Japanese study noted a worse prognosis for Kras mutant patients undergoing resection of CLM [17]. Kras mutation was an independent predictor of worse oncologic end points including disease specific survival (DSS) (P=0.006, HR: 2.86), time to surgical failure (P<0.001 HR: 2.42), recurrence free survival (RFS) (P=0.048; HR: 1.47), liver RFS (P=0.026, HR: 1.67), and lung RFS (P<0.001 HR: 2.56). Similar to our study, where smaller ablation margins were a predictor of shorter time to new liver metastases, a positive surgical resection margin was an important predictor of liver RFS (P<0.001, HR: 3.5) [17]. Kras mutation was independently associated with shorter DSS after colon resection (P=0.03 HR: 1.9) and after CLM resection (P=0.001, HR: 2.4) [16]. Shorter OS (52.2% versus 81%, P=0.002) and RFS (13.5% versus 33.5%, P=.001) for Kras mutant patients after surgical resection of CLM were also documented in a different cohort [14]. In the latter, lung RFS was significantly shorter at 3 years (34.6% versus 59.3%, P<0.001) although liver RFS at 3 years did not reach significance (43.8% versus 50.2%, P=0.181) [14]. A different group similarly noted that Kras mutation was independently associated with shorter OS (P=0.02, HR: 1.65) [18]. Although there was no difference in the RFS and pattern of recurrences for the Kras mutant patients, within the sub-group of patients who experienced a recurrence those with a Kras mutation had significantly shorter survival (P=0.002, HR: 2.07) [18] indicating that recurrences in Kras mutant patients carry a more aggressive disease biology. A recent meta-analysis of 8 studies concluded that Kras mutation was associated with worse OS (HR: 2.24) and RFS (HR: 1.89) after resection of CLM [19]. Recently, a study showed a significantly lower survival and recurrence free survival for Kras mutant patient with CLM undergoing percutaneous ablation (microwave or radiofrequency) [23].

An interesting finding in our study was the significantly higher LTP rate (almost double) for mutant Kras tumors versus the wild type ablated CLM with margin of 1-5 mm. The LTP rate for Kras mutant tumors of 1-5 mm was similar to tumors with no ablation margin and the risk of progression was 15.6-fold more that of wild type tumors with margins ≥6 mm. A recent retrospective study noted that the proportion of positive resection margins (<1 mm) for CLM was significantly higher for RAS mutant patient at 11.4% versus 5.4% for wild type patients (P=0.008), and this retained significance on multivariate analysis (P=0.005; HR: 2.4) [24]. The authors indicated that one of the possible explanations could be a more infiltrative tumor biology in the mutant RAS tumor type [24]. Infiltrative histological type of CLM was associated with a higher incidence of liver recurrences after resection in another study; 38.9% versus 16.2% (P=0.02) [25]. In that study the percentage of resection margins of 1-5 mm was slightly higher for the infiltrative than the pushing type (42.6% versus 29.7%) [25]. This further highlights the importance of achieving sufficient ablation margins >5 mm [8, 9, 26] particularly for the Kras mutant patients. In addition to the general poor prognostic value of the KRAS mutation, our findings of higher LTP for the KRAS mutant vs wild type tumors ablated with suboptimal margin suggest an underlying relation between KRAS mutant tumors and resistance to heat. This is concordant with prior investigations indicating a dependence of heat shock protein expression in CRC on KRAS mutation and PI3K activation at least in colon cancer cell lines [27]. Prior investigations have also indicated a relation between heat stress and PI3K/mTORC2-dependent AKT signaling that may be a mediator of liver cancer survival after thermal ablation [28]. This further highlights the need to achieve larger radiographic ablation margins especially for the KRAS mutant patients. Ideally margins should be 10 mm all over the target tumor as this offers the best possible local tumor control [4, 26] however a minimum of 6 mm margin is absolutely required especially for the Kras mutant tumors.

This study has several limitations. We only included mutation in Codon 12 and 13 of the Kras gene in our analysis. Extended RAS mutational analysis has shown that the exon 2 mutations are the most frequent (89%) with exon 3 and 4 mutations occurring in 7% of cases and the remaining 4% of cases having an Nras mutation [22]. The study suffers the limitations of the retrospective analysis conducted in a single center as well as the small number that did not allow meaningful analysis of entire genome and mutational profile.. The impact of systemic chemotherapy post-RFA on disease control and OS was not analyzed in this study. Similarly, the impact of post-RFA hepatic arterial chemotherapy for patient with liver recurrences post-ablation was not analyzed. Another limitation is the lack of histopathologic correlation to verify complete tumor destruction after ablation. In a recent prospective study evaluation of complete necrosis by histopathology and immunohistochemistry combined with sufficient ablation margins offered the best possible local tumor control (97%) after RFA of CLM [9].

In conclusion, Kras mutation is a marker of shorter overall survival, and shorter time to new liver and peritoneal metastases after RFA of CLM. There is a trend for a higher cumulative incidence of LTP and shorter time to new lung metastases in the Kras mutant group. It is essential to create an ablation margin of at least 6 mm and ideally 10 mm (A0) surrounding the tumor in general and particularly in the Kras mutant patients in order to provide the best possible local tumor control.

## METHODS

### Study population

A retrospective review of our CLM RFA database was performed under a waiver from our Institutional Review Board, from December 2002 through April 2013. Patients with documented Kras mutational status were included. The study population consisted of 97 consecutive patients with 148 CLM ablated in 120 sessions (median CLM size: 1.7, range: 0.6 – 5 cm) with 1-3 tumors ablated per session.

Radiographic evidence of extrahepatic disease (EHD) was documented in 47 patients (48%) at the time of RFA. This included lung only (n=32), lung and other site (n=13) (LN and/or peritoneum), or 1 site outside the lung (n=2) (LN or pelvic implant). These patients were classified into two groups: lung only (n=32) and >1 site/other site (n=17).

### Genetic marker mutation analysis

The specimens used for analysis originated from resected liver metastases (in 57 patients), the primary tumor (in 29 patients), or other metastatic sites (in 11 patients). Formalin-fixed paraffin embedded tissue was used to extract DNA samples for analysis. Kras exon 2 (codon 12 and 13) mutations were analyzed by Sanger sequencing using an ABI 3730 DNA sequencer [15, 22, 29]. Mass-spectrometry assay (Sequenom, San Diego, CA) was used for extended mutation analysis of Kras, Nras, EGFR, ERBB2, Braf, PIK3CA, MEK1, and AKT1 [15, 22, 29].

### Ablation procedure

Image guided percutaneous ablation was performed under general anesthesia in all cases [8, 26]. PET/CT guidance was used in 7 sessions (6%) and split-dose PET/CT guidance in 25 sessions (21%) as previously described [30]. We used the following ablation devices: Covidien Cool Tip electrode (n=96) (Covidien, Mansfield, Mass), RITA XL/XLI electrode (n=43) (Angiodynamics, Latham, NY), LeVeen electrode (n=9) (Boston Scientific, Natick, Mass). Ablations were completed as per manufacturers’ protocol with the aim of achieving an ablation margin ≥5 mm all around the target tumor.

### Imaging follow-up and definitions

#### Follow-up regimen

The first post-ablation imaging was performed with contrast enhanced CT (CECT) at 4-8 weeks to assess complete tumor eradication (Technique effectiveness). This was used as the baseline for future comparisons. Subsequent CECT examinations every 2-4 months continued to monitor hepatic or extra-hepatic recurrences. Additionally, MRI and/or FDG-PET/CT were performed for equivocal findings on CECT.

#### Imaging and definitions

We used the guidelines of the Society of Interventional Radiology (SIR) for terminology and reporting [31]. Local tumor progression (LTP) at the site of the ablation was defined on the portal venous phase of CECT as any peripheral/nodular enhancement or any hypodense lesion(s) at or within 10 mm of the ablation zone [31, 32]. For FDG-PET (PET/CT), LTP was defined as any PET avid focus near or at the ablation margin as judged by Nuclear medicine faculty. New liver tumors were defined on the portal venous phase of CECT or on FDG-PET (PET/CT) as any tumor >1cm from the ablation zone. Additionally we recorded the dates of site-specific recurrences (lung, peritoneum, bone, and brain) for patients without involvement of those sites at the time of ablation.

Technique effectiveness was defined as complete coverage of the target tumor by the ablation zone/defect at the first (4-8 weeks) post ablation CT scan. Overall survival (OS) was defined as the time from the first RFA till death or latest follow-up. LTP-free survival (LTPFS) was defined as the time from RFA till imaging evidence of LTP or latest follow-up. Time to new liver, lung, peritoneal, bone, and brain metastases was defined as the time from RFA till the imaging evidence of new metastases at those sites.

#### Ablation margin

Ablation margin was measured as previously described [8, 26]. This was done on a PACS workstation using the portal venous phase of the CECT prior to RFA and the 1^st^ 4-8 weeks CECT post-RFA [8, 26]. Ablated CLM were classified as having no (0 mm) margin, 1-5 mm margin, 6-10 mm margin, or >10 mm margin (A0). The ablation margin could be measured for 113/148 tumors (75%) and 75/97 patients (77%). It could not be measured if: margin difficult to accurately define (n=19; fused ablation defects), tumor not visible on CECT (n=10), only MRI or PET/CT available on 1^st^ follow-up scan (n=5), or if the 1^st^ follow-up scan was more than 8 weeks post-ablation (n=1).

### Statistical analysis

Kras exon 2 mutational analysis was available for all patients. Because of the low rate of mutations for the other markers, only Kras exon2 mutation was evaluated as a predictor of outcomes. Additional clinical and technical factors evaluated as predictors of outcomes included: sex, age >60, node positive primary, disease-free interval >12 months, tumor number >1, history of prior liver resection, prior hepatic arterial infusion chemotherapy (HAIC), tumor size >3cm, CEA > 30 ng/ml, extra-hepatic disease, and ablation margin size. Survival probabilities were estimated using the Kaplan-Meier method. A chi-square test was used to compare the proportions of new liver, lung, and peritoneal metastases between the mutant and wild type patients.

Univariate analysis (UV) was performed using a log-rank test and multivariate analysis using a Cox-regression model for OS. A competing risk model (Fine and Gray) was employed for UV and multivariate analysis (MV) of LTP-free survival (LTPFS), time to new liver, lung, and peritoneal metastases. The model was adjusted for the effect of clustering for LTPFS in patients with >1 CLM ablated [33]. A backward-stepwise selection was employed for MV including variables with a P-value <0.15 on UV. The competing risk analysis enables us to isolate the risk of local tumor progression by modeling separately the subdistribution functions of LTP as well as death without LTP and the hazard rates from these subdistributions (called subhazards).

Cumulative incidence function was used to calculate the cumulative incidence at 1, 2, 3 years for LTP at the ablation site and site specific recurrence for new liver, lung, peritoneum, bone, and brain metastases.

Statistical significance was defined as two-sided P-value <0.05. All statistical analysis was done using STATA version 12.0 (StataCorp LP, College Station, Texas). The cumulative incidence of LTP was calculated using the cmprsk package using the software R version 3.2.3.
